# Clinical Validation of a CRX Variant Leading to a Cone-Rod Dystrophy

**DOI:** 10.7759/cureus.103528

**Published:** 2026-02-13

**Authors:** Camila Pagán-Melvin, Natalio Izquierdo, Karla C Alejandro

**Affiliations:** 1 Ophthalmology, Universidad Central del Caribe, Bayamon, PRI; 2 Ophthalmology, University of Puerto Rico School of Medicine, Medical Sciences Campus, San Juan, PRI

**Keywords:** cone-rod dystrophy, crx gene, genotype-phenotype correlation, inherited retinal dystrophy, variant reclassification

## Abstract

Patients with cone-rod dystrophy (CORD) due to *CRX* mutations have progressive visual impairment characterized by central vision loss, photophobia, and color vision defects. On ophthalmic examination, patients with CRX-associated CORD may have macular abnormalities, changes in the retinal pigment epithelium, and progressive macular degeneration, affecting central visual function.

Variants in the *CRX* gene located on chromosome 19q13 lead to photoreceptor dysfunction and retinal degeneration. Variant classification in *CRX* presents unique challenges, as approximately half of heterozygous missense variants may be benign, requiring careful phenotype-genotype correlation for accurate pathogenicity determination.

Our patient had progressive vision loss, bilateral macular abnormalities, and visual symptoms compatible with CORD. The patient's clinical findings included central visual field defects, reduced multifocal electroretinography responses, and preserved full-field electroretinography consistent with macular-restricted disease.

Next-generation sequencing showed a heterozygous, likely pathogenic variant c.166G>A (p.Ala56Thr) located within the homeodomain at residue 56. Comprehensive ophthalmic examination, electrophysiological testing, and genetic studies may all help reach a CORD diagnosis. This case highlights the importance of phenotype-driven variant interpretation for reclassifying *CRX* variants from "likely pathogenic" to "pathogenic" and raises clinical awareness for the critical role of genotype-phenotype concordance in inherited retinal dystrophies.

## Introduction

Inherited retinal diseases (IRDs) are a clinically and genetically heterogeneous group of disorders that lead to progressive vision loss and include a subgroup of cone and cone-rod dystrophies (CORDs). The prevalence of IRDs is estimated at 1 in 1,043 [1], with CORD specifically estimated to affect 1 in 40,000 individuals [2]. 

CORD is characterized by a predominant degeneration of cones affecting the macula initially [2], followed by rod involvement. However, there are limited cases where there is a simultaneous involvement of rods and cones. Initial presentation, as early as the first decade of life, can include decreased visual acuity, color vision defects, and severe photophobia [2]. In later stages of disease, impaired night vision becomes evident, and loss of peripheral vision progresses, often resulting in legal blindness [2].

Non-syndromic CORDs are genetically heterogeneous and inherited in a Mendelian pattern. Multiple genes have been implicated in CORD, with inheritance patterns including autosomal-dominant, autosomal-recessive, and X-linked forms. The *CRX* gene is one of the major contributors to CORD and is almost always inherited in an autosomal-dominant pattern [3]. The *CRX* gene, located on chromosome 19, encodes the cone-rod homeodomain protein expressed in photoreceptors, retinal pigment epithelium (RPE) cells, and the pineal gland [3]. This protein acts as a transcription factor in the regulation of photoreceptor expression, differentiation, development, and maintenance [4]. The location of pathogenic variants within the *CRX* gene correlates with distinct clinical phenotypes. Mutations affecting the homeodomain region predominantly result in CORD or macular dystrophy characterized by bull’s eye maculopathy, while variants located downstream of the homeodomain are associated with a broader phenotypic spectrum, including Leber congenital amaurosis (LCA), CORD and macular dystrophy, and retinitis pigmentosa [5].

According to Hamel [2], diagnosis of CORD is clinical, based on the presence of early decrease in visual acuity and photophobia, macular lesions, and/or retinal atrophy upon ophthalmoscopy, and reduced-amplitude ERG traces suggesting cone involvement. Genetic testing plays a central role in confirming the diagnosis of CORD and identifying the specific causative variant. However, variant interpretation presents significant challenges, as novel or rare variants may be classified as variants of uncertain significance (VUSs) or likely pathogenic based solely on computer-based prediction tools and population frequency data. Clinical phenotype-genotype correlation is essential for reclassifying variants and establishing definitive pathogenicity. 

We report a case of CORD in a patient found to have a heterozygous c.166G>A (p.Ala56Thr) variant within the homeodomain region (residue 56) of the *CRX* gene. This variant demonstrates supportive in silico prediction scores (SIFT: 0.23, PolyPhen2: 0.96) [6] and is currently classified as likely pathogenic. The patient’s clinical presentation of CORD aligns precisely with the expected phenotype for homeodomain missense mutations, providing strong evidence for reclassifying this variant as pathogenic.

## Case presentation

A 65-year-old female patient was referred to our clinics for ophthalmic genetics consultation due to progressive visual impairment. The patient complained of a gradual vision decline over several years, affecting primarily central visual function, photophobia, and dyschromatopsia.

Upon comprehensive ophthalmic evaluation by one of the authors (NJI), best-corrected visual acuity was found to be 20/50 and 20/40 in the right eye and left eye, respectively. Pupillary examination revealed pupils equal, round, and reactive to light, without a relative afferent pupillary defect. Intraocular pressure (IOP) was 13 mmHg in both eyes. Slit-lamp examination demonstrated nuclear sclerotic cataracts bilaterally. 

**Figure 1:**
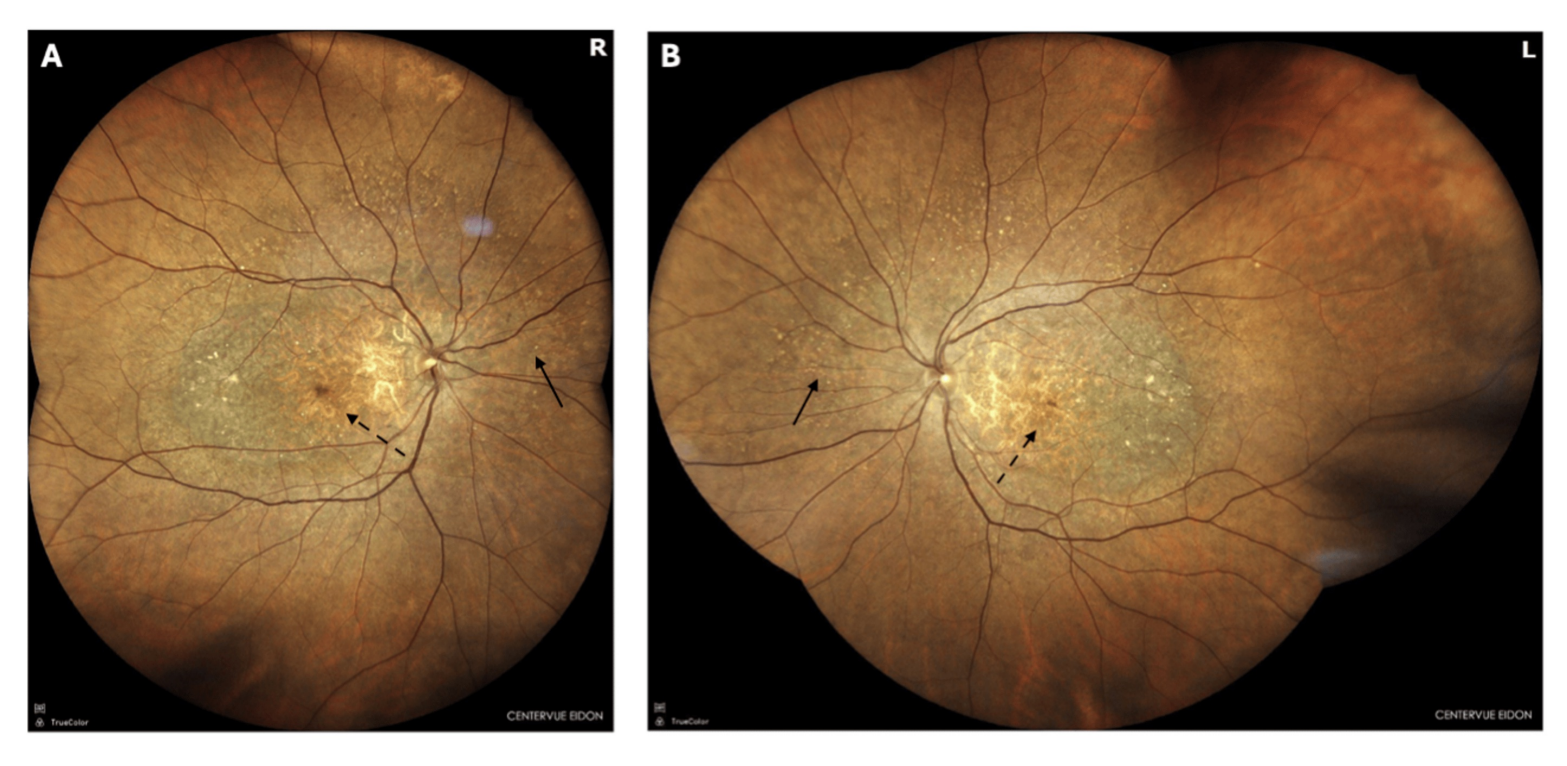
Color fundus photography showing bilateral macular degeneration (A) Right eye shows geographic atrophy (dashed arrows), retinal pigment epithelium mottling and hypertrophy (solid arrows), chorioretinal degeneration, and fundus tessellation in the macula. (B) Left eye shows similar findings with bilateral macular pigmentary changes (solid arrows) and atrophy (dashed arrows).

As shown in Figure 1, ophthalmoscopic examination revealed bilateral macular abnormalities, including geographic atrophy, RPE hypertrophy, macular and chorioretinal degeneration, and fundus tessellation. Cup-to-disc and artery-to-vein ratios were within normal limits in both eyes. 

**Figure 2:**
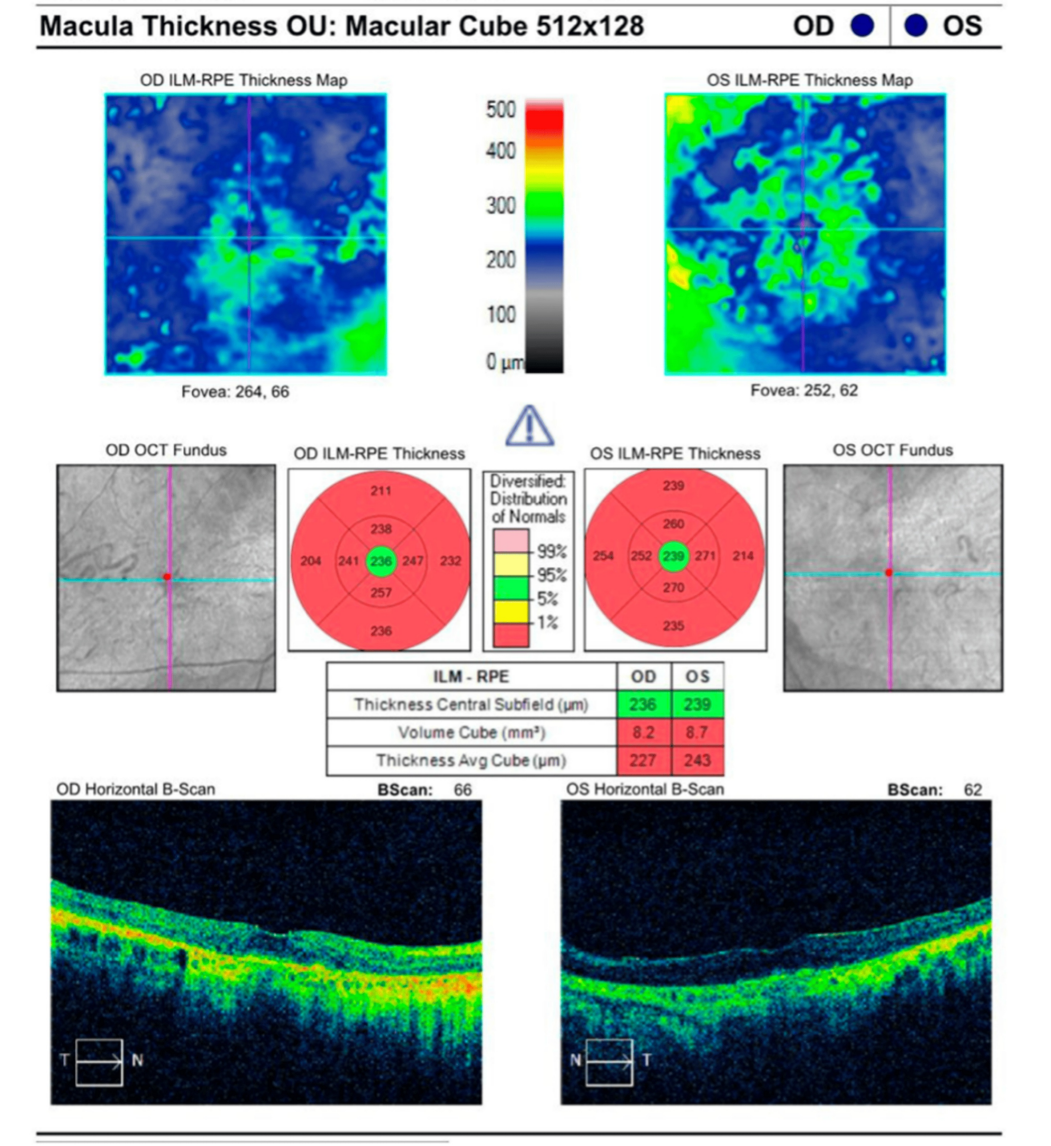
OCT of the macula showing bilateral outer retinal layer attenuation Conventions: The normative color scale indicates values within normal limits (green: 5th-95th percentile), borderline (yellow: 1st-5th percentile), and outside normal limits (red: <1st percentile) compared to the device's age-matched database. OCT, optical coherence tomography; ILM, inner limiting membrane; RPE, retinal pigment epithelium.

Optical coherence tomography (OCT) examination revealed bilateral macular thinning with attenuation of the outer retinal layers (Figure 2). No intraretinal or subretinal fluid was observed. 

**Figure 3:**
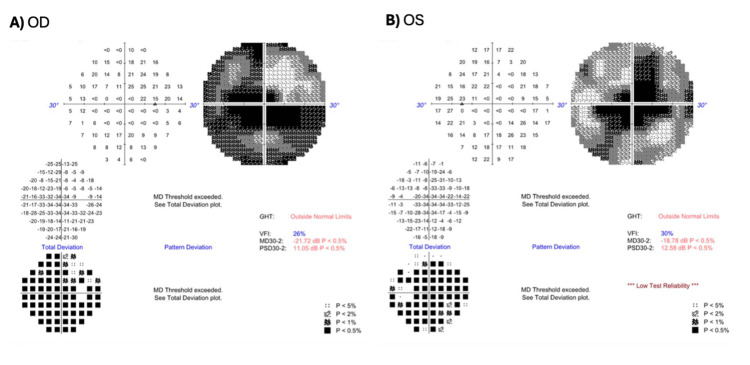
Visual field testing (30-2) showing bilateral central scotomas A) Right eye (OD) shows a significantly decreased MD of -21.72 dB (p < 0.5%) and a PSD of 11.05 dB (p < 0.5%) with a VFI of 26%. (B) Left eye (OS) shows a significantly decreased MD of -18.78 dB (p < 0.5%) and a PSD of 12.58 dB (p < 0.5%) with a VFI of 30%. GHT is outside normal limits bilaterally. GHT, glaucoma hemifield test; VFI, visual field index; MD, mean deviation; PSD, pattern standard deviation.

**Figure 4:**
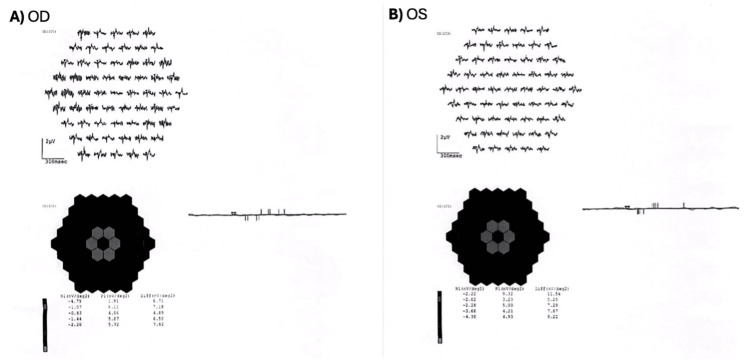
mfERG showing bilateral central retinal dysfunction (A) Right eye (OD) showing severely reduced central responses in rings R1-R3 with marked attenuation of response amplitudes. (B) Left eye (OS) showing reduced central responses in rings R1-R3 with relatively better preservation compared to the right eye. The 3D response density plots demonstrate central depression with lighter (gray) hexagons indicating reduced response amplitudes, with abnormal quantitative ring-response indices bilaterally. mfERG, multifocal electroretinography.

Automated Humphrey visual field testing demonstrated bilateral central sensitivity loss (Figure 3). Full-field electroretinography (ffERG) showed preserved global function, with normal photopic and scotopic response amplitudes and mildly delayed latencies. In contrast, multifocal electroretinography (mfERG) confirmed reduced central responses in the central rings R1-R3, OD < OS, with abnormal quantitative ring-response indices (Figure 4). 

Given the discordance between preserved ffERG responses and abnormal mfERG findings, genetic testing was ordered. Next-generation sequencing with the Invitae Inherited Retinal Disorders Panel was done (Laboratory for Molecular Medicine, Center for Genetics and Genomics, Cambridge, MA). It identified a heterozygous *CRX* variant (c.166G>A; p.Ala56Thr), classified as likely pathogenic. Additional heterozygous VUSs were identified in the *LRP5* and *ROM1* genes. 

## Discussion

Previous studies have established distinct clinical features and age-of-onset patterns for *CRX*-associated CORD. According to Gill and co-workers [7], the classic triad presentation of CORD leads to predominant central vision loss, photophobia, and color vision disturbance. Fujinami-Yokokawa and co-workers [8] reported that *CRX*-associated CORD typically presents with late-onset disease, with a median age of onset at 45 years. Importantly, they noted that heterozygous missense variants within the homeodomain underlie the mild CORD phenotype with late-onset disease, often presenting in the sixth decade [8]. Our patient, at age 65 years, had a gradual decline in vision over several years, primarily affecting central vision function. She complained of photophobia and poor color vision. This presentation aligns with the classic triad of CORD and late-onset pattern typical of *CRX*-associated disease. The presence of these symptoms, combined with her age at presentation, is consistent with the phenotype associated with homeodomain variants, which can manifest as a gradual decline [5,8]. Considering the clinical features reported in the literature, including late-onset presentation and variable symptomatology, our patient’s presentation correlates with the phenotypic expression of CORD.

Ocular findings in patients with CORD include macular dystrophy characterized by progressive atrophy of the RPE and neuroretina, predominantly affecting the macula and midperiphery, with funduscopic areas of degeneration [6]. According to Kim and co-workers [5], the macular-centered distribution sparing the peripheral retina is pathognomonic for homeodomain variants, characteristically distinguishing it from more widespread retinal disease occurring in downstream variants. Upon ophthalmoscopic examination, our patient revealed bilateral macular abnormalities, including geographic atrophy, RPE hypertrophy, macular and chorioretinal degeneration, and increased visibility of the underlying choroidal vessels. These findings are consistent with the macular-predominant pattern characteristic of *CRX* homeodomain variants, confirming the expected phenotype for CORD-causing variants. 

OCT findings in patients with CORD show characteristic patterns of photoreceptor and RPE degeneration. Lima and co-workers [9] demonstrated that patients with CORD consistently showed a complete absence of the interdigitation zone across the entire macular scan, including the foveal area, reflecting abnormal outer retinal morphology with potential absence of photoreceptor outer segment or defective interdigitation between the apical processes of the RPE and outer segments of the cone. Additionally, the external limiting membrane and the ellipsoid zone have shown to be lost in most patients [9]. Furthermore, studies have found a decreased central retinal thickness consistent with photoreceptor loss in patients with *CRX* truncation mutations [10]. Our patient’s OCT revealed bilateral macular thinning with attenuation of the outer retinal layers, including loss of the ellipsoid zone and external limiting membrane, and preservation of the inner retinal layers. The optic nerve and retinal vasculature appeared normal. The normal optic nerve finding further supports the diagnosis, since *CRX*-associated disease primarily affects photoreceptors and RPE. The pattern of outer retinal layer loss with preserved inner retinal architecture and normal optic nerves is consistent with the photoreceptor-specific degeneration found in CORD. 

Central visual field defects are the earliest and most predominant finding in CORD, with decreased sensitivity in the central visual field reflecting primary cone involvement and macular-centered pathology [2]. Visual field testing typically reveals progressive constriction, with central scotomas appearing first, followed by gradual loss of peripheral sensitivity. Thiadens and co-workers [11] found that 70% of CORD patients showed peripheral field defects 10 years after diagnosis, distinguishing CORD from pure cone dystrophy. Most importantly, Kim and co-workers [5] described that variants within the homeodomain presented with CORD or macular dystrophy with bull’s eye maculopathy, suggesting that visual field defects in these patients would be predominantly macular-centered with relative peripheral sparing, at least in early stages. Automated visual field testing in our patient demonstrated bilateral dense central scotomas with marked central sensitivity loss and relatively preserved peripheral fields. This pattern is consistent with the macular-restricted disease of *CRX* homeodomain variants, rather than the widespread peripheral involvement seen in later stages of disease or in downstream variants.

The pattern of central visual field loss observed in our patient necessitated further electrophysiological evaluation to characterize the extent of photoreceptor dysfunction. ffERG often remains preserved in early or macular-restricted *CRX*-associated disease [12]. mfERG is particularly sensitive to localized central cone dysfunction in CORD and is used to follow disease progression [13]. Findings in the ffERG in our patient demonstrated preserved global function, with normal photopic and scotopic response amplitudes and mildly delayed latencies. In contrast, mfERG revealed reduced central responses in the central rings R1-R2, OD < OS, with abnormal quantitative ring-response indices. The discordance between ffERG and mfERG directly supports macular-restricted CORD. Our patient’s electrophysiological pattern is entirely consistent with the macular dystrophy seen in *CRX* homeodomain variants in CORD. This visual field loss pattern, combined with the funduscopic findings of macular-centered degeneration and the electrophysiologic pattern of predominant cone dysfunction on mfERG, provides robust evidence supporting the pathogenicity of the p.Ala56Thr variant. 

According to Yi and co-workers [14], even though half of the heterozygous missense variants may be benign, pathogenic variants within the homeodomain cause autosomal-dominant retinal degeneration. On the other hand, Fujinami-Yokokawa and co-workers [8] demonstrated that patients with homeodomain variants displayed a characteristic CORD phenotype with progressive central vision loss, with these heterozygous missense variants specifically underlying the mild CORD phenotype with late-onset disease, often in the sixth decade. Our patient’s genetic evaluation showed a heterozygous mutation in the *CRX* gene with a c.166G>A (p.Ala56Thr) variant located within the homeodomain region at residue 56 of the *CRX* gene. According to the American College of Medical Genetics and Genomics and the Association for Molecular Pathology (ACMG/AMP) guidelines [15], multiple criteria support the pathogenicity of the CRX c.166G>A (p.Ala56Thr) variant. The variant is currently classified as likely pathogenic in ClinVar (Variation ID: 99596) [16], with Labcorp Genetics noting that "additional data are needed to prove that conclusively." Our case provides this additional phenotypic evidence. The variant satisfies PM1 as it is located within the critical homeodomain region at residue 56, a well-established mutational hotspot for pathogenic CRX variants. PM2 applies as the variant is extremely rare in population databases (gnomAD frequency 0.004%, rs61748437). PP3 is met through multiple in silico prediction tools indicating deleterious effects (SIFT:0.23, PolyPhen2: 0.96) [4]. PP4 applies as the patient's phenotype is highly specific for CRX-associated disease, which follows autosomal-dominant inheritance with high penetrance for homeodomain variants. The ACMG/AMP emphasizes the importance of phenotype matching as critical evidence for variant interpretation [17]. When a patient shows clinical features associated with the disease spectrum for a specific gene, the variant should be considered pathogenic. Together, these criteria (PM1, PM2, PP3, PP4) provide strong cumulative evidence supporting the reclassification of this variant from likely pathogenic to pathogenic. 

In contrast, variants downstream of the homeodomain are associated with earlier-onset, severe diseases like LCA. The p.Ala56Thr variant’s location therefore predicts the observed non-LCA phenotype with later-onset macular involvement, which precisely aligns with our patient’s presentation at 65 years of age. It is important to note that ABCA 4-associated Stargardt disease was considered in the differential diagnosis due to similar macular-restricted degeneration with central atrophy and peripheral sparing. However, the genetic panel confirmed the absence of pathogenic variants in ABCA4, ruling out Stargardt disease and supporting the CRX variant as the causative mutation. When a VUS or likely pathogenic classification demonstrates complete phenotypic alignment with the known disease spectrum, including age of onset, patterns of visual loss, structural changes, and functional deficit, this provides objective in vivo evidence of pathogenicity. 

Being a sole case, our findings are limited and cannot establish broader genotype-phenotype patterns or assess phenotypic variability of this specific variant. Family members were not available for genetic testing, precluding segregation analysis to confirm the autosomal-dominant inheritance pattern within this family or to assess penetrance. Additionally, this specific variant has limited reported cases in population databases, and population frequency data may be incomplete, which restricts comparative analysis with other affected individuals carrying the same variant. 

This case report provides clinical evidence, following the ACMG/AMP PP4 criterion, supporting the reclassification of the *CRX* c.166G>A (p.Ala56Thr) variant from “likely pathogenic” to “pathogenic.” Our patient’s clinical presentation demonstrates phenotypic alignment with the expected disease spectrum for *CRX* homeodomain variants, including late-onset macular-restricted CORD, central visual field loss, macular degeneration with peripheral sparing, outer retinal layer loss on OCT, and preserved ffERG with reduced mfERG responses. This case contributes essential phenotypic data for this specific variant and reinforces the importance of detailed clinical characterization in establishing variant pathogenicity for inherited retinal dystrophies. 

## Conclusions

Clinical diagnosis of inherited retinal dystrophies remains challenging due to overlapping phenotypes. CORD associated with *CRX* mutations must remain part of the differential diagnosis when evaluating patients with progressive central vision loss and macular abnormalities. Inheritance patterns in patients with *CRX*-associated retinal dystrophies have been described, with homeodomain variants following autosomal-dominant inheritance. Comprehensive ophthalmic examination, including fundoscopy, OCT, mfERG, and genetic studies, may all contribute to reaching a definitive CORD diagnosis. Prompt diagnosis may help in genetic counseling, prognostic assessment, and monitoring disease progression in affected patients and their families. Although genetic testing is an increasingly helpful tool to aid in diagnosing and managing these patients, technological and interpretive shortcomings, such as "VUS" and "likely pathogenic" classifications requiring clinical validation, should be considered. Pathogenicity determination for *CRX* variants is particularly complex, as a significant proportion of heterozygous missense mutations may represent benign variants. Both the specific location within the gene and the nature of the variant must be carefully evaluated, with homeodomain missense variants demonstrating pathogenicity only when supported by concordant clinical phenotypes.

Our patient, who had a heterozygous *CRX* c.166G>A (p.Ala56Thr) variant within the homeodomain and a clinical CORD diagnosis with complete genotype-phenotype concordance, represents an important contribution to variant reclassification: demonstrating objective clinical evidence that supports upgrading this variant from "likely pathogenic" to "pathogenic" classification. This case reinforces phenotype-driven variant interpretation as essential for accurate phenotypic and genotype pathogenicity assessment. 
